# Investigating the biochemical response of proton minibeam radiation therapy by means of synchrotron-based infrared microspectroscopy

**DOI:** 10.1038/s41598-024-62373-9

**Published:** 2024-05-25

**Authors:** Roberto González-Vegas, Ibraheem Yousef, Olivier Seksek, Ramon Ortiz, Annaïg Bertho, Marjorie Juchaux, Catherine Nauraye, Ludovic De Marzi, Annalisa Patriarca, Yolanda Prezado, Immaculada Martínez-Rovira

**Affiliations:** 1 Physics Department, Universitat Autònoma de Barcelona (UAB), Campus UAB Bellaterra, 08193 Cerdanyola del Vallès, Spain; 2 MIRAS Beamline BL01, ALBA-CELLS Synchrotron, Cerdanyola del Vallès, 08209 Barcelona, Spain; 3 IJCLab, French National Centre for Scientific Research, 91450 Orsay, France; 4 Institut Curie, CNRS UMR3347, Inserm U1021, Signalisation Radiobiologie et Cancer, Institut Curie, Université PSL, Orsay, France; 5 Radiation Oncology Department, Institut Curie, INSERM LITO, PSL Research University, University Paris-Saclay, Campus Universitaire, 91898 Orsay, France; 6 CNRS UMR3347, Inserm U1021, Signalisation Radiobiologie et Cancer, Université Paris-Saclay, 91400 Orsay, France; 7 New Approaches in Radiotherapy Lab, Center for Research in Molecular Medicine and Chronic Diseases (CIMUS), Instituto de Investigación Sanitaria de Santiago de Compostela (IDIS), University of Santiago de Compostela, 15706 Santiago de Compostela, A Coruña, Spain; 8 Oportunius Program, Galician Agency of Innovation (GAIN), Xunta de Galicia, Santiago de Compostela, A Coruña, Spain

**Keywords:** Radiotherapy, Radiotherapy

## Abstract

The biology underlying proton minibeam radiation therapy (pMBRT) is not fully understood. Here we aim to elucidate the biological effects of pMBRT using Fourier Transform Infrared Microspectroscopy (FTIRM). In vitro (CTX-TNA2 astrocytes and F98 glioma rat cell lines) and in vivo (healthy and F98-bearing Fischer rats) irradiations were conducted, with conventional proton radiotherapy and pMBRT. FTIRM measurements were performed at ALBA Synchrotron, and multivariate data analysis methods were employed to assess spectral differences between irradiation configurations and doses. For astrocytes, the spectral regions related to proteins and nucleic acids were highly affected by conventional irradiations and the high-dose regions of pMBRT, suggesting important modifications on these biomolecules. For glioma, pMBRT had a great effect on the nucleic acids and carbohydrates. In animals, conventional radiotherapy had a remarkable impact on the proteins and nucleic acids of healthy rats; analysis of tumour regions in glioma-bearing rats suggested major nucleic acid modifications due to pMBRT.

## Introduction

Radiotherapy (RT) is one of the most important cancer treatment options, as approximately 50% of the patients suffering this disease will be treated with RT at some point during the course of their illness. This modality has benefited over the last decades from significant technological advances, resulting in greater efficiencies in dose conformation at the tumour site and reduced toxicities to healthy tissues. Nonetheless, some challenging hurdles are yet to be overcome, such as the treatment of high-grade gliomas due to their inherent radioresistances; using higher doses is usually not an option due to the limitations imposed by nearby organs at risk, in order to avoid undesirable side effects.

Spatially fractionated radiation therapy (SFRT) is a modality based on the use of several beamlets that was proposed to address these limitations. The result is a widening of the therapeutic window, with increased dose tolerance in healthy tissues. Depending on the chosen beam characteristics (width and spacing), there are several SFRT modalities, such as GRID therapy, LATTICE therapy, minibeam radiation therapy (MBRT) or microbeam radiation therapy (MRT)^1^. This study will focus on MBRT, a modality that employs arrays of 0.5–1.0 mm-wide beamlets spaced a center-to-center (c-t-c) distance of approximately 1–4 mm apart. MBRT represents a compromise in terms of beam width between GRID/LATTICE therapy (1–2 cm) and MRT (50–100 $\upmu$m), but the beams are narrow enough to preserve the tissue-sparing effect^2^. In addition, the beam characteristics of MBRT allow its low-cost implementation in numerous facilities, unlike MRT which is limited to synchrotrons. Several studies reported remarkable results using MBRT, such as no significant brain lesions and reduced skin damage^2^, high tumour control^3^ and fewer long-term toxicities^4^, all compared to broad beam (BB) irradiations.

Diverse radiation types (photons, protons, carbon ions, etc.) and energies have been explored in MBRT^4,5^. Among all available options, the most widely investigated combination has been proton minibeam radiation therapy (pMBRT)^6^. The use of proton beams in combination with SFRT offers a series of advantages over other particles, such as the absence of dose deposition after the Bragg peak, and the possibility to obtain homogeneous dose distributions at the tumour site while preserving the minibeam pattern at shallower depths. In addition, several preclinical studies on pMBRT have shown remarkable results compared with conventional proton RT, demonstrating fewer undesirable side effects and suggesting an equal or superior tumour control and survival rate, both in the short and long-terms^7,8^. However, a complete picture of the biological processes underlying pMBRT is still lacking, although some mechanisms such as differential effects on immature vessels^9^ or immune activation as antitumour response^10^ have been proposed.

Within this context, the present study aims to provide new insights into the subjacent biology of pMBRT using Fourier Transform Infrared Microspectroscopy (FTIRM), analysing the effects of irradiations in healthy and tumour cell lines, as well as in healthy and tumour-bearing rats. FTIRM has proven to be a powerful, non-destructive tool for examining biological samples^11^. The measured infrared (IR) absorbance spectra can reveal detailed information about the biochemical structure and the possible conformational modifications undergone by the samples. FTIRM has previously helped to assess cell and tissue responses to various treatments, including RT^12–14^. To the best of our knowledge, this is the first study to report biochemical insights, both for in vitro and in vivo pMBRT irradiations, using FTIRM.

## Materials and methods

### In vitro studies: sample preparation and irradiations

CTX-TNA2 rat astrocytes and F98 rat glioma cell lines (ATCC^®^-CRL-2006 and ATCC^®^-CRL-2397, respectively) were purchased from LGC Standards (Molsheim, France). Both cell populations were standardly cultured in high glucose (4.5 g/L) Gibco™DMEM medium (Life Technologies SAS, Courtaboeuf, France) supplemented with 10% fetal calf serum, 1% penicillin–streptomycin (10,000 units/mL each), 1 mM GlutaMAX™, 1 mM sodium pyruvate and 10 mM HEPES. Incubation was performed in a chamber at 37 ^∘^C, 95% humidity and 5% CO_2_. Prior to irradiation, cells were trypsinized and counted; a volume of 1 mL of cell suspension was seeded in each well of 24-well microplates at a concentration of 5 $\times$ 10^4^ cells/mL in order to reach a 75% confluence rate on the day of RT after an overnight incubation. Cells were directly grown onto 0.5 mm-thick IR transparent calcium fluoride (CaF_2_) coverglasses (Crystran Ltd) placed on the bottom of wells.

Irradiations were performed at the pencil beam scanning beamline of the Institut Curie Proton Therapy Center (ICPO, Orsay, France). It uses an “universal” nozzle-equipped gantry supplied by a Proteus 235 isochronous cyclotron (IBA, Belgium) capable of delivering both pencil beam scanning and double scattering treatment modalities. The proton beam energy was 100 MeV. A divergent 6.5 cm-thick multislit brass collimator was attached to the nozzle exit. It consisted of 15 slits with a width of 400 $\upmu$m and a c-t-c distance of 4 mm. The distance between the collimator exit and the cells was 7 cm, while the media thickness was 10 mm. Radiochromic films were placed on the cell slides to ensure irradiation quality and differentiate the peak and valley dose regions.

Three different mean doses ${\overline{D}}$ were employed for each cell line: 2, 5 and 10  Gy for astrocytes, and 5, 10 and 20  Gy for glioma cells; in all cases, the peak-to-valley dose ratio (PVDR) was around 10, with the following peak and valley doses: 6.5 ± 0.3 Gy and 0.70 ± 0.05 Gy (${\overline{D}}$ = 2.1 ± 0.1 Gy), 15.2 ± 0.8 Gy and 1.6 ± 0.1 Gy (${\overline{D}}$ = 5.2 ± 0.3 Gy), 32 ± 1 Gy and 3.0 ± 0.2 Gy (${\overline{D}}$ = 10.0 ± 0.5 Gy), and 64 ± 3 Gy and 5.5 ± 0.3 Gy (${\overline{D}}$ = 19.8 ± 0.9 Gy). The linear energy transfer (LET) values at the cell layer, which were estimated by Monte Carlo simulations, were 1.2 keV/$\upmu$m in the peak regions and 2.6 keV/$\upmu$m in the valley regions^15^. The doses for glioma cells were higher than those for astrocytes, due to the superior radiation-resistant properties of the F98 cell line^16^.

One day after irradiations, the coverglasses were rinsed with phosphate-buffered saline (PBS) and incubated for 1 h at room temperature in 10% formalin neutral buffered solution (Sigma-Aldrich). Then, after 3 rounds of rinsing with ultrapure water to wash out any residual phosphate ions, the samples were dried for FTIRM measurements, following previous protocols^17,18^.

Metabolic activity post-RT was also evaluated using the resazurin-resorufin assay. The details and results of the assay are included in the Supplementary Information.

### In vivo study: animals, tumour inoculation and irradiations

All animal experimental protocols were approved by the French Ministry of Research (permit number 2019122418442057). All methods were carried out in accordance with relevant guidelines and regulations on animal welfare and ethical guidelines at the Institute Curie. All methods reported are in accordance with ARRIVE guidelines.

Six-week-old male immunocompetent rats (Fischer 344) were acquired from Janvier Labs. Brain tissue analysis was performed at 2 h and 24 h post-RT in healthy rats, and at 24 h post-RT for tumour-bearing rats. For the latter animals, the F98 (ATCC^®^-CRL-2397) glioma cell line was transfected with the luciferase gene. F98-Luc cells (10,000) were suspended in 5 $\upmu$L of DMEM and then injected intracranially using a Hamilton syringe through a burr hole in the right caudate nucleus (− 1 mm anterior-posterior, 4 mm median-lateral, and − 5.5 mm dorsal–ventral distances from the skull). Bioluminescence imaging (BLI) was done using an IVIS spectrum (PerkinElmer, Houten, the Netherlands) to confirm tumour presence 1 day prior to irradiation (14 days after F98 implantation), as described in previous works^19^.

For each configuration, two animals per irradiation modality (BB and pMBRT) were submitted to RT (whole brain irradiations, excluding the cerebellum and olfactory bulbs), plus two control rats. The prescribed ${\overline{D}}$ was 30 Gy at 1.6 mm-depth (tumour position of F98-bearing rats). For proton BB irradiations, a 16 mm-wide collimator was employed. Regarding pMBRT, a similar collimator to that used for cell irradiations was employed, but with 5 slits separated by a c-t-c distance of 2.8 mm. Radiochromic films were placed on the rat heads for dosimetry assessment; peak and valley doses were 59 ± 2 Gy and 14.5 ± 1.0 Gy, respectively.

Right after brain extraction, they were snap-frozen and stored at − 80 ^∘^C. 5 $\upmu$m-thick sagittal sections were cut from the right side of the brains (to match the entrance of MBs) and deposited onto low-e microscope slides (Kevley Technologies), suitable for FTIRM measurements. Sections were immersed in zinc formalin solution (Sigma-Aldrich) and then, rinsed with ultrapure water and dried. Staining of samples was not possible after sample preparation for FTIRM measurements.

### FTIRM measurements at ALBA synchrotron

Both cells and tissue samples were submitted to FTIRM measurements at the MIRAS beamline of ALBA-CELLS Synchrotron (Cerdanyola del Vallès, Spain). IR spectra were acquired using the Hyperion 3000 microscope coupled to the Vertex 70 spectrometer (Bruker Optics GmbH, Germany) using a mercury cadmium telluride liquid nitrogen-cooled detector.

The synchrotron beam was used for the in vitro study, ensuring a good signal-to-noise ratio in single cell measurements (aperture size of 8 $\times$ 8 $\upmu$m^2^). Between 100 and 150 cells were randomly selected from each sample and irradiation configuration (control, BB, MB_peak_, MB_valley_). Single point maps of the individual cells were collected in the 3800–900 cm^-1^ spectral range, with a spectral resolution of 4 cm^-1^; 128 co-added scans were recorded per spectrum. Background spectra (256 co-added scans) were also collected every 10 cells in a sample-free region. Regarding tissue measurements, a conventional IR source was employed. IR raster scanning maps (100 $\times$ 100 $\upmu$m^2^ spacing) of the whole rat brain sections were collected for each sample and irradiation condition with 4 co-added scans per spectrum.

### FTIRM data analysis

Data was analysed using the Quasar software version 1.7 (https://quasar.codes)^20^. Principal Component Analysis (PCA) was employed as a dimensionality reduction method to investigate differences in spectral features. The analysis was divided into two distinct spectral regions: the higher wavenumber region (HW; 3000–2800 cm^-1^), dominated by the stretching modes of methylene and methyl groups related to the hydrocarbon chain length of lipids^21^; and the amides + fingerprint regions (A+FP; 1800–950 cm^-1^), consisting of peaks representative of the proteins, with two main bands named Amide I (AI, 1710–1598 cm^-1^) and Amide II (AII, 1590–1483 cm^-1^) arising from C=O-stretching, NH-bending and CN-stretching modes^22^, as well as the fingerprint region (FP region; 1350–950 cm^-1^) with contributions from the carbohydrates and phosphates (Phosphate I, PhI, 1270–1186 cm^-1^; Phosphate II, PhII, 1146–1004 cm^-1^) of the nucleic acids^11^.

Prior to PCA, the Savitzky–Golay filter (second derivative order; 17 points window in the A+FP region, 11 points window in the HW region) and unit vector normalization were applied to the cells IR spectra. Violin plots were constructed to assess the probability density of various spectral ratios of interest, used as markers of biochemical modifications^14,23^.

In addition, hyperspectral images of the whole tissue sections were generated to evaluate the distribution of specific spectral ratios. The areas under the corresponding spectral bands were obtained after applying a baseline correction to the raw data.

## Results and discussion

### CTX-TNA2 and F98 cell studies

**Figure 1:**
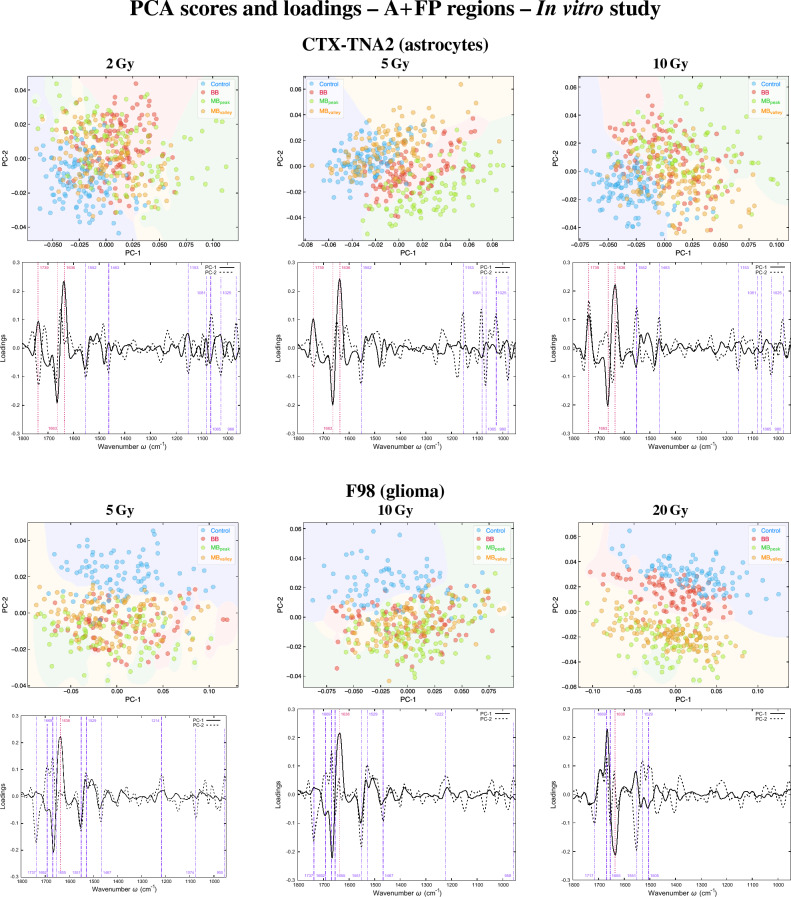
In vitro study. PCA in the A+FP regions of CTX-TNA2 (astrocytes, top) and F98 (glioma, bottom) cells irradiated with the indicated doses; for each cell line, the PCA scores (upper row) and loadings (lower row) are included. Explained variances by the PCs are about 45% and 55% for the healthy and tumour rat cell lines, respectively. Each point of the PCA scores represents a cell spectrum, and colours correspond to the irradiation configurations: blue for control (non-irradiated), red for BB, green for MB_peak_ and orange for MB_valley_. Vertical lines in the loadings indicate the most relevant peaks for each PC: dashed and pink for PC-1, and dot-dashed and purple for PC-2. The mean doses for BB and pMBRT irradiations were 2, 5 and 10 Gy for astrocytes, and 5, 10 and 20 Gy for glioma cells. For pMBRT, the specific peak and valley doses were 6.5 ± 0.3 Gy and 0.70 ± 0.05 Gy (${\overline{D}}$ = 2.1 ± 0.1 Gy), 15.2 ± 0.8 Gy and 1.6 ± 0.1 Gy (${\overline{D}}$ = 5.2 ± 0.3 Gy), 32 ± 1 Gy and 3.0 ± 0.2 Gy (${\overline{D}}$ = 10.0 ± 0.5 Gy), and 64 ± 3 Gy and 5.5 ± 0.3 Gy (${\overline{D}}$ = 19.8 ± 0.9 Gy). MBs were generated by means of a divergent collimator of 15 slits with a width of 400 $\upmu$m, separated a c-t-c distance of 4 mm.

**Figure 2:**
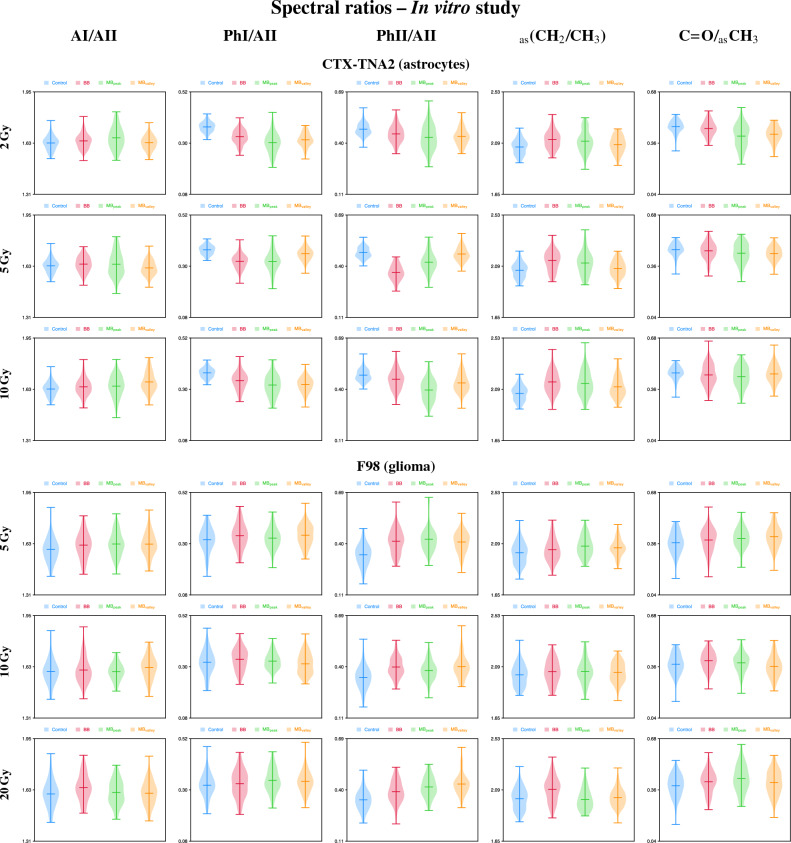
In vitro study. Intensity distributions of the (from left to right) AI/AII, PhI/AII, PhII/AII, _as_(CH_2_/CH_3_) and C=O/_as_CH_3_ spectral ratios of CTX-TNA2 (astrocytes, top) and F98 (glioma, bottom) cells. Each row corresponds to one dose. Irradiation modalities are coloured in blue for control, red for BB, green for MB_peak_ and orange for MB_valley_. The mean doses for BB and pMBRT irradiations were 2, 5 and 10 Gy for astrocytes, and 5, 10 and 20 Gy for glioma cells. For pMBRT, the specific peak and valley doses were 6.5 ± 0.3 Gy and 0.70 ± 0.05 Gy (${\overline{D}}$ = 2.1 ± 0.1 Gy), 15.2 ± 0.8 Gy and 1.6 ± 0.1 Gy (${\overline{D}}$ = 5.2 ± 0.3 Gy), 32 ± 1 Gy and 3.0 ± 0.2 Gy (${\overline{D}}$ = 10.0 ± 0.5 Gy), and 64 ± 3 Gy and 5.5 ± 0.3 Gy (${\overline{D}}$ = 19.8 ± 0.9 Gy). MBs were generated by means of a divergent collimator of 15 slits with a width of 400 $\upmu$m, separated a c-t-c distance of 4 mm.

**Figure 3:**
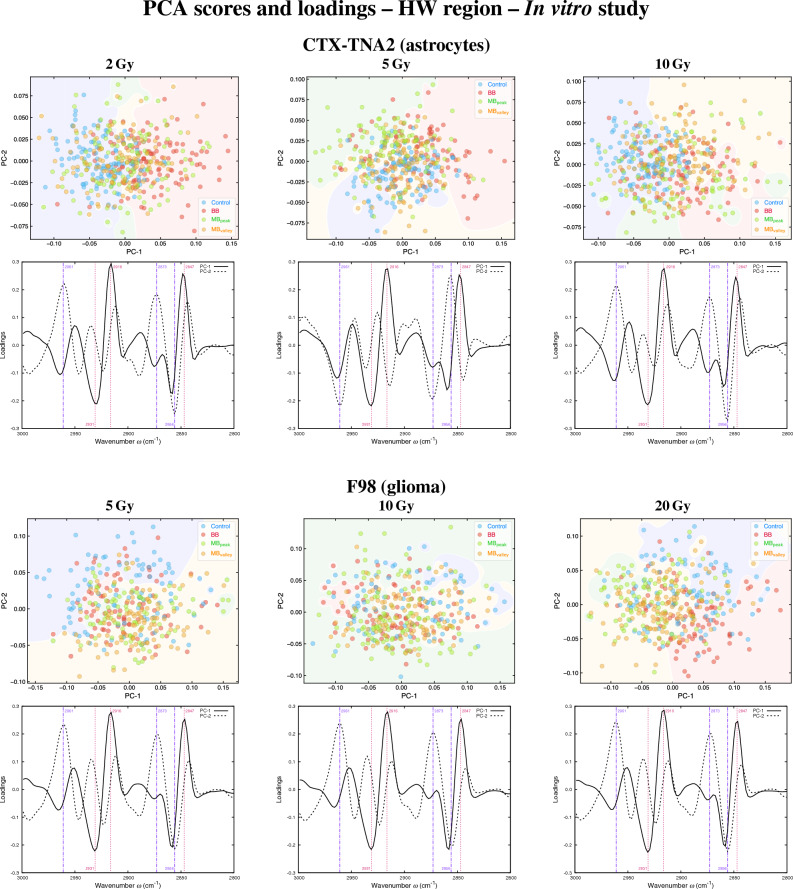
In vitro study. PCA in the HW region of CTX-TNA2 (astrocytes, top) and F98 (glioma, bottom) irradiated with the indicated doses; for each cell line, the PCA scores (upper row) and loadings (lower row) are included. Explained variances by the PCs are about 85% for both cell lines. Each point of the PCA scores represents a cell spectrum, and colours correspond to the irradiation configuration: blue for control (non-irradiated), red for BB, green for MB_peak_ and orange for MB_valley_. Vertical lines in the loadings indicate the most relevant peaks for each PC: dashed and pink for PC-1, and dot-dashed and purple for PC-2. The mean doses for BB and pMBRT irradiations were 2, 5 and 10 Gy for astrocytes, and 5, 10 and 20 Gy for glioma cells. For pMBRT, the specific peak and valley doses were 6.5 ± 0.3 Gy and 0.70 ± 0.05 Gy (${\overline{D}}$ = 2.1 ± 0.1 Gy), 15.2 ± 0.8 Gy and 1.6 ± 0.1 Gy (${\overline{D}}$ = 5.2 ± 0.3 Gy), 32 ± 1 Gy and 3.0 ± 0.2 Gy (${\overline{D}}$ = 10.0 ± 0.5 Gy), and 64 ± 3 Gy and 5.5 ± 0.3 Gy (${\overline{D}}$ = 19.8 ± 0.9 Gy). MBs were generated by means of a divergent collimator of 15 slits with a width of 400 $\upmu$m, separated a c-t-c distance of 4 mm.

The principal components (PCs) scatter plots of both astrocytes and glioma are presented in Fig. 1 (A+FP regions) and Fig. 3 (HW region), along with the loading plots which helped to identify the most relevant spectral features (wavenumbers) contributing to data separation, previously multiplied by − 1^24^. The violin plots showing the probability density of selected spectral ratios for both cell lines are depicted in Fig. 2. The results of the cytotoxicity assay are included in the Supplementary Information (Fig. S1).

#### Amides + fingerprint regions

For astrocytes (Fig. 1, top), a marked separation between the four irradiation configurations is observed. MB_peak_ and BB groups separate from non-irradiated cells along both PC-1 and PC-2, being the MB_valley_ group closer to control samples.

The loadings revealed that separation of groups along PC-1 is mainly due to modifications of the AI. The main peaks affected arise at 1663 cm^-1^ and 1636 cm^-1^, assigned to the $\alpha$-helix and $\beta$-sheet structures, respectively. Alterations in these structures could indicate conformational changes following irradiations, which might be related to protein oxidation mechanisms^25^, particularly due to the BB and MB_peak_ groups for the intermediate and high doses. Differences in the amides could also indicate protein modifications due to nucleic acid repairing processes^26^. Another relevant peak located at 1739 cm^-1^ originates from the carbonyl ester C=O stretching modes of lipids^27^. Modifications in this band have been reported in cells becoming mainly non-hydrogen bonded and loosing membrane integrity^28^, or with the lipids of cells suffering free radical attacks due to oxidative stress^29^.

Further differences between doses can be detected by inspecting the loadings of PC-2. Apart from the carbonyl ester and the substructures of AI, a peak located at 1552 cm^-1^ is common for the three doses, assigned to the $\alpha$-helix of AII^12^. Bending modes of the CH_2_ group also arise at 1464 cm^-1^^22^. Modifications of these bands could also suggest conformational changes in the proteins secondary structure upon RT^12^, especially due to the BB and MB_peak_ for the intermediate and high doses.

Specific peaks in the FP region contribute to data separation mainly along PC-2. The band at 1153 cm^-1^ arise from stretching vibrations of hydrogen-bonding C–OH groups in the nucleic acids and carbohydrates, and its modification suggests adjustments of these groups in the DNA, seen during early apoptotic cell death^30^. The region 1080–1065 cm^-1^ includes the PhII band, which arises from symmetric stretching vibrations of the phosphodiester group of the nucleic acids, and the stretching vibration modes of the C–O bond of the ribose; double strand breaks (DSBs) might have caused the modifications of these bands^31,32^. The peak around 1025 cm^-1^ corresponds to stretching vibrations of the furanose C–O group, and may indicate alterations of the DNA and RNA structures^12^. Overall, a different impact on the nucleic acids and carbohydrates of the BB and MB_peak_ configurations comparing with the control and MB_valley_ groups is seen. Along these lines, clear differences can be observed in the PhI/AII and PhII/AII spectral ratios (Fig. 2). Both ratios exhibit a reduction of the values for irradiated groups compared to control cells, especially for the MB_peak_ group. A decrease of phosphate absorbances was observed upon oxidative stress^33^ and might be indicative of a more tightly packed DNA^12,34^. Also, a reduction in these bands has been correlated with conformational changes and/or rearrangements of the nucleic acids, due to an increase of single strand breaks (SSBs), DSBs and base cleavage reactions with dose^30,35^; hydrogen bonding-adjustments may have also occurred during cell death processes^35^. Other studies did also observe this reduction upon an early detection of nuclear DNA modifications before complete fragmentation^34^. All these effects seem to be more important for BB irradiations and the pMBRT peak regions. In addition, the trends of the PhI/AII and PhII/AII spectral ratios seem to be the most correlated with the expressed cytotoxicity (Fig. S1).

Additionally, bands in the 980–960 cm^-1^ spectral region of the PC-2 loadings are common for the three doses of astrocytes. These peaks are assigned to vibrations of the phosphates and the deoxyribose^27^, and have been observed upon SSBs and DSBs of single and double-stranded DNA after proton irradiations^36^; according to data separation, these changes would have been induced mainly by the BB and MB_peak_ groups.

Group separation for glioma cells (Fig. 1, bottom) occurs between control and all irradiated configurations. For the highest dose, pMBRT groups are well apart from control and BB samples. This separation occurs mainly along PC-2, as well as along PC-1 for the intermediate and highest doses. The main peaks contributing to data separation in PC-2 are related to the AI, the carbonyl ester, the $\alpha$-helix of AII and the CH_2_ bending modes; these bands were also relevant for astrocytes and may indicate similar biochemical processes. Additionally, two peaks arise at 1529 cm^-1^ and 1505 cm^-1^, assigned to $\beta$-sheets of AII^37^ and to CH in-plane bending^27^, respectively; these are especially relevant for the highest dose. Regarding PC-1, the $\beta$-sheet of AI is highly contributing to data separation, and may indicate secondary structure protein modifications due to oxidative stress or cell death processes, particularly for the highest dose of pMBRT.

Bands in the FP region also contribute to data separation, especially for the low and intermediate doses. The PhI peak around 1220–1210 cm^-1^ is assigned to a secondary structure of the DNA (B-DNA)^38^, and could indicate RNA modifications^32^. Another band at 955 cm^-1^ arises from deoxyribose vibrations and might indicate SSBs or DSBs in DNA^36^. Analysing the PhI/AII and PhII/AII spectral ratios (Fig. 2), the three irradiation configurations experienced an intensity increase compared to control cells; for the highest dose, the intensities of the pMBRT groups are also higher than for BB. Therefore a different biochemical behavior is observed in tumour cells compared to healthy cells. Previous studies also observed an increase of these ratios for a human prostate adenocarcinoma cell line upon proton irradiations, as a result from strand cleavage and chromatin fragmentation due to increased DNA breakages^31^. A higher intensity of the PhI was also correlated with cells being under oxidative stress^39^.

#### Higher wavenumber region

Figure 3 shows the PCA in the HW region of astrocytes and glioma. Few differences between clusters are observed for the three doses, with irradiated groups slightly separating from control samples in both cell lines. For astrocytes, the mild separation of the irradiated groups occurs mainly along PC-1, and the loadings show that the peaks with major contributions correspond to the CH_2_ asymmetric stretching (2931 cm^-1^) and to asymmetric and symmetric C-H stretching vibrations of the CH_2_ groups of fatty acids (2916 cm^-1^ and 2847 cm^-1^)^40^. For F98 cells, the slight separation between non-irradiated and irradiated groups occurs along both PCs, with the main peaks affected being the CH_3_ asymmetric stretching (2961 cm^-1^), the CH_3_ symmetric stretching (2873 cm^-1^), and the CH_2_ symmetric stretching (2856 cm^-1^).

The asymmetric methylene to asymmetric methyl _as_(CH_2_/CH_3_) spectral ratio (2945–2900 cm^-1^ and 2980–2945 cm^-1^, respectively), related to modifications in the acyl chain length of lipids^14,21^, was also assessed. Figure 2 shows an increase in the ratio for the BB and MB_peak_ configurations compared to control cells for all doses of astrocytes, being less pronounced for the MB_valley_. For tumour cells, an increase of this ratio is only observed for the BB group and the highest dose. Such increases may indicate that cells exhibit longer lipid chains under oxidative stress^41,42^. Also, an increase in this ratio could be indicative of cell death processes due to irradiations^31^.

The overall modifications in lipids, along with the alterations in proteins and nucleic acids described in the previous section, are consistent with a superior oxidative stress of BB and MB_peak_ groups with respect to MB_valley_. Recent Monte Carlo studies reported differences in the primary yields of various reactive oxygen species (ROS) between BB and MB peaks compared to MB valleys, resulting in the low-dose regions inducing less damage due to greater ROS recombination than in the high-dose regions^43,44^, in agreement with our results.

### Healthy and tumour-bearing rat brain sections

**Figure 4:**
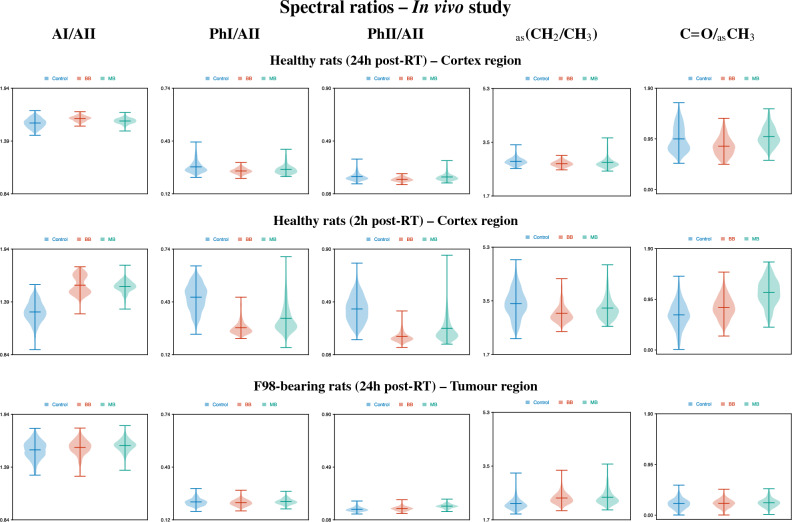
In vivo study. Violin plots assessing the probability density of the (from left to right) AI/AII, PhI/AII, PhII/AII, _as_(CH_2_/CH_3_) and C=O/_as_CH_3_ spectral ratios of healthy rat brain sections 24 h (top) and 2 h (middle) post-RT in the cortex region, and of F98-bearing rat brain section 24 h post-RT in the tumour region (bottom). The colours indicate the irradiation configuration: control in blue, BB in red and MB in green. The mean dose for BB and pMBRT irradiations was 30 Gy. For pMBRT, the specific peak and valley doses were 59 ± 2 Gy and 14.5 ± 1.0 Gy. MBs were generated by means of a divergent collimator of 5 slits with a width of 400 $\upmu$m, separated a c-t-c distance of 2.8 mm.

Figures S2, S3 and S4 (Supplementary Information) include the hyperspectral images showing the distribution of the spectral ratios for the healthy (at 24 h and 2 h post-RT) and tumour-bearing (at 24 h post-RT) rat brain sections, respectively. Figure 4 shows the violin plots assessing the probability density of the same spectral ratios: for the healthy rats, the cortex region was considered to construct the plots, while for the F98-bearing rats, the tumour region was selected.

At 24 h post-irradiations, there is an increase in the AI/AII ratio in the cortex region of healthy rat brains, particularly for the BB configuration; this can be indicative of the BB modality inducing greater modifications in the proteins secondary structure than pMBRT for this particular dose (30 Gy). As for the PhI/AII and PhII/AII ratios, intensities decreased for the irradiated groups compared to control animals, especially for BB-treated rats; this trend is akin to the results for astrocytes in the in vitro study. A reduction in the _as_(CH_2_/CH_3_) ratio was also noted for the two RT modalities, which may be a result of free radical attacks on phospholipid membranes during oxidative stress^33^. In addition, the BB-irradiated tissues are associated with a small reduction in the C=O/_as_CH_3_ spectral ratio relative to the other groups.

In order to assess the early response to radiation, healthy tissue analysis was also performed at 2 h post-RT. The biochemical modifications at this time-point are larger than at 24 h post-RT, especially in the proteins and nucleic acids regions. Most of the pMBRT-induced damage observed at 2 h post-RT seem to be repaired at 24 h post-RT. Figure S3 and Fig. 4 show a large increase in the AI/AII spectral ratio for both irradiated groups compared to control animals. Proteins are known to play an important role in the repair of radiation-induced nucleic acids lesions^45^. Therefore, the increased intensity of the AI and AII bands may be due to an up-regulation of proteins involved in brain repair processes upon RT^26,46^. Conversely, a marked decrease of the PhI/AII and PhII/AII spectral ratios indicating an increased nucleic acids radiation damage was observed in RT-treated rats, particularly for BB irradiations. Regarding lipid bands, a decrease of the _as_(CH_2_/CH_3_) ratio for irradiated configurations is similar to the one observed at 24 h post-RT. Instead, an increase in the C=O/_as_CH_3_ is clearly seen at 2 h post-RT, especially for pMBRT, which could be correlated with oxidative stress and to ROS damage^33^.

The hyperspectral images of the tumour-bearing rats (Fig. S4) revealed significant biochemical differences between the tumours and the surrounding healthy tissues. For the AI/AII ratio, the intensity in the tumours slightly increased compared to the nearby normal tissue. Contrarily, the rest of the ratios are less intense in the tumours of the brain maps. A lower amount of nucleic acids in malignant (ovarian cancer) with respect to normal tissue was previously reported^47^. The reduction of the _as_(CH_2_/CH_3_) intensity in the tumours may be ascribed to differences in the oxidative environment of the malignancy due to glucose degradation and lactic acid production^48^.

Concerning the variations among irradiation configurations in tumour-bearing rats, the violin plots in Fig. 4 show an increase of the PhII/AII ratio for the pMBRT-treated rats compared to the other groups, which is consistent with the in vitro results for the F98 cells and may indicate enhanced nucleic acid damage. An increase of the _as_(CH_2_/CH_3_) ratio can also be observed, but this time of both irradiated groups relative to the control animals, suggesting similar modifications of hydrocarbon chain lengths induced by both types of treatment.

## Conclusions

For the first time, this work evaluated the biochemical modifications in CTX-TNA2 rat astrocytes and F98 rat glioma cell lines subjected to conventional proton RT and pMBRT, as well as in healthy and tumour-bearing animals, using FTIRM.

The PCA for astrocytes in the A+FP revealed modifications in the proteins spectral region. Such alterations were particularly relevant for the BB and MB_peak_ groups, suggesting conformational changes in the structure of proteins as a result of irradiations. The effect of these two groups also appears to be important in the FP region, which is related to the nucleic acids and carbohydrates: numerous bands assigned to the phosphodiester groups were affected. Along with an examination of the PhI/AII and PhII/AII spectral ratios, possible adjustments or rearrangements of the nucleic acids structure may have occurred as a result of an increase of DSBs or oxidative stress. According to the PCA, the MB_valley_ group was always closer to control samples than the other two irradiation configurations, demonstrating a different biochemical impact on cells than BB and MB_peak_ regions. The analysis of the HW region revealed subtle changes in the CH_x_ stretching modes due to RT modalities. Regarding the F98 cell line, similar conformational modifications as seen for astrocytes appear to have occurred. The cluster separation among irradiated configurations in the FP region is less pronounced than for healthy cells, as most of the peaks accounting for data separation arose in the proteins spectral region. In the tumour cell line, contrary to the healthy cell line, the BB groups are closer to control, and MB_valley_ and MB_peak_ groups overlap in almost all configurations. The trend of the PhII/AII spectral ratio correlated with nucleic acid damage for all irradiation configurations.

In vivo hyperspectral imaging analysis revealed the biochemical pattern in the whole brain for the several irradiation configurations. The spectral ratios of healthy rat cortex fixated at 24 h post-RT revealed conformational modifications in the proteins and nucleic acids, mainly due to BB irradiations. These modifications were more pronounced 2 h after RT. Most of the pMBRT-induced damage observed in healthy tissue at 2 h post-RT seem to be repaired at 24 h post-RT.

Regarding the F98-bearing animals, indications of enhanced DNA damage due to pMBRT were associated with an increase of the PhII/AII spectral ratio. There were also signs of hydrocarbon chain modifications after irradiations due to an increase of the _as_(CH_2_/CH_3_) ratio for both RT modalities compared to untreated animals.

This FTIRM study provided, for the first time, new insights into the biochemical effects involved in pMBRT, and encourages the use of IR microspectroscopy to further investigate novel RT approaches.

### Supplementary Information

Supplementary Information.

## Data Availability

Research data will be stored and made available in an institutional repository (https://dataverse.csuc.cat).
