# Supplementary material for: Investigating the biochemical response of proton minibeam radiation therapy by means of synchrotron-based infrared microspectroscopy
Source: Sci Rep. 2024 May 25;14:11973. doi: 10.1038/s41598-024-62373-9 (PMC11128026; doi:10.1038/s41598-024-62373-9)
Supplement: Supplementary file 1 — Supplementary Information. [file 41598_2024_62373_MOESM1_ESM.pdf]

Supplementary Information

Investigating the biochemical response of proton minibeam radiation therapy by means of synchrotron-based infrared microspectroscopy

Roberto González-Vegas[1], Ibraheem Yousef[2], Olivier Seksek[3], Ramon Ortiz[4,6], Annaïg Bertho[4,6], Marjorie Juchaux[4,6], Catherine Nauraye[5], Ludovic De Marzi[5], Annalisa Patriarca[5], Yolanda Prezado[4,6,7,8], and Immaculada Martínez-Rovira[1,*]

[1]*Physics Department, Universitat Autònoma de Barcelona (UAB), 08193 Bellaterra, Cerdanyola del Vallès, Barcelona, Spain*

[2]*MIRAS Beamline BL01, ALBA-CELLS Synchrotron, 08209 Cerdanyola del Vallès, Barcelona, Spain*

[3]*IJCLab, French National Centre for Scientific Research, 91450 Orsay, France*

[4]*Institut Curie, CNRS UMR3347, Inserm U1021, Signalisation Radiobiologie et Cancer, Institut Curie, Université PSL, Orsay, France*

[5]*Radiation Oncology Department, Institut Curie, PSL Research University, University Paris-Saclay, INSERM LITO, Campus Universitaire, 91898 Orsay, France*

[6]*Université Paris-Saclay, CNRS UMR3347, Inserm U1021, Signalisation Radiobiologie et Cancer, 91400 Orsay, France*

[7]*New Approaches in Radiotherapy Lab, Center for Research in Molecular Medicine and Chronic Diseases (CIMUS), Instituto de Investigación Sanitaria de Santiago de Compostela (IDIS), University of Santiago de Compostela, 15706 Santiago de Compostela, A Coruña, Spain*

[8]*Oportunius Program, Galician Agency of Innovation (GAIN), Xunta de Galicia, Santiago de Compostela, A Coruña, Spain*

[*]Corresponding author. E-mail address: Immaculada.Martinez@uab.cat.

Cytotoxicity assay

A day after irradiations, cells were harvested in the wells of the 24-well plate by using the trypsin-EDTA procedure. After counting, 100 µL of 10^5 cells/mL suspension in fresh medium were seeded in 96-well microplates. One treated well of the 24-well plate allowed to seed 3 to 5 wells of the 96-well plate. Cells were then incubated for 24 hours at 37 °C, 95% humidity and 5% CO_2. Subsequently, medium was replaced by 100 µL of fresh medium and 10 µL of alamarBlue™ HS Cell Viability Reagent solution (Invitrogen) were added. Cells were incubated for 2 hours at 37 °C and the fluorescence intensity (FI) measurement (575 nm excitation, 590 nm emission) of each well was obtained using a Fluoroskan Ascent FL microplate reader (Thermo Fisher Scientific, Illkirch, France). The percentage of the metabolic activity (or growth inhibition) of the cell populations was evaluated by calculating the ratio of the FI after treatment to the control FI.

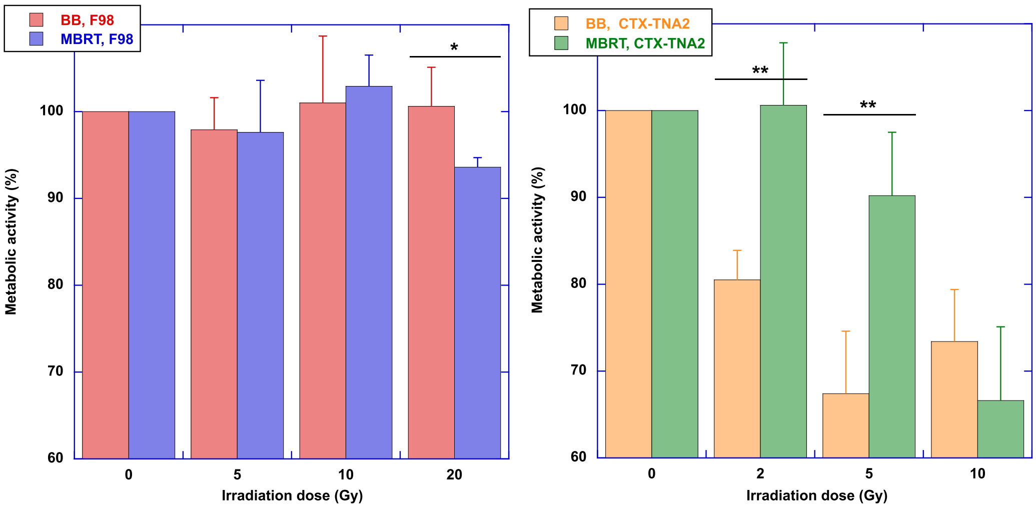

Figure S1. Metabolic activity of F98 (glioma, left) and CTX-TNA2 (astrocytes, right) cell lines. Red and orange boxes are BB-irradiated cells, whereas blue and green boxes are pMBRT-treated cells. Statistically significant differences between groups with p-values lower than 0.05 (*) and 0.01 (**) are indicated. The mean doses for BB and pMBRT irradiations were 2, 5 and 10 Gy for astrocytes, and 5, 10 and 20 Gy for glioma cells. For pMBRT, the specific peak and valley doses were 6.5 ± 0.3 Gy and 0.70 ± 0.05 Gy ($\overline{D}$ = 2.1 ± 0.1 Gy), 15.2 ± 0.8 Gy and 1.6 ± 0.1 Gy ($\overline{D}$ = 5.2 ± 0.3 Gy), 32 ± 1 Gy and 3.0 ± 0.2 Gy ($\overline{D}$ = 10.0 ± 0.5 Gy), and 64 ± 3 Gy and 5.5 ± 0.3 Gy ($\overline{D}$ = 19.8 ± 0.9 Gy). MBs were generated by means of a divergent collimator of 15 slits with a width of 400 µm, separated a c-t-c distance of 4 mm.

Unlike FTIRM analysis, it was not possible to differentiate measurements between pMBRT peaks and valleys: the values obtained are therefore a combination of the differential effects on the entire cell population. The results shown in Fig. S1 indicate a difference in radiosensitivity between the two cell types. Indeed, from 0 to 20 Gy, percentages remain significantly constant at around 100% viability for F98, whatever the type of irradiation. For 0 to 10 Gy, the CTX-TNA2 cell line adopts a different behavior: sensitive to BB for the 2 Gy (80.5 ± 3.4%), and less sensitive to MBRT for 2 and 5 Gy (100 ± 7.2% and 90.2 ± 7.3%, respectively) but reaching a similar cytotoxicity at 10 Gy whatever the modality (73.4 ± 6.0% and 66.6 ± 8.5% for BB and pMBRT, respectively). This is probably due to the nature of these cells and to their ability to react to radiation damage. On this latter point, among the measurements obtained by FTIRM, the trends of the PhI/AII and PhII/AII spectral ratios seem to be the most correlated with expressed cytotoxicity (Fig. 2 of the main manuscript).

In vivo study: hyperspectral images

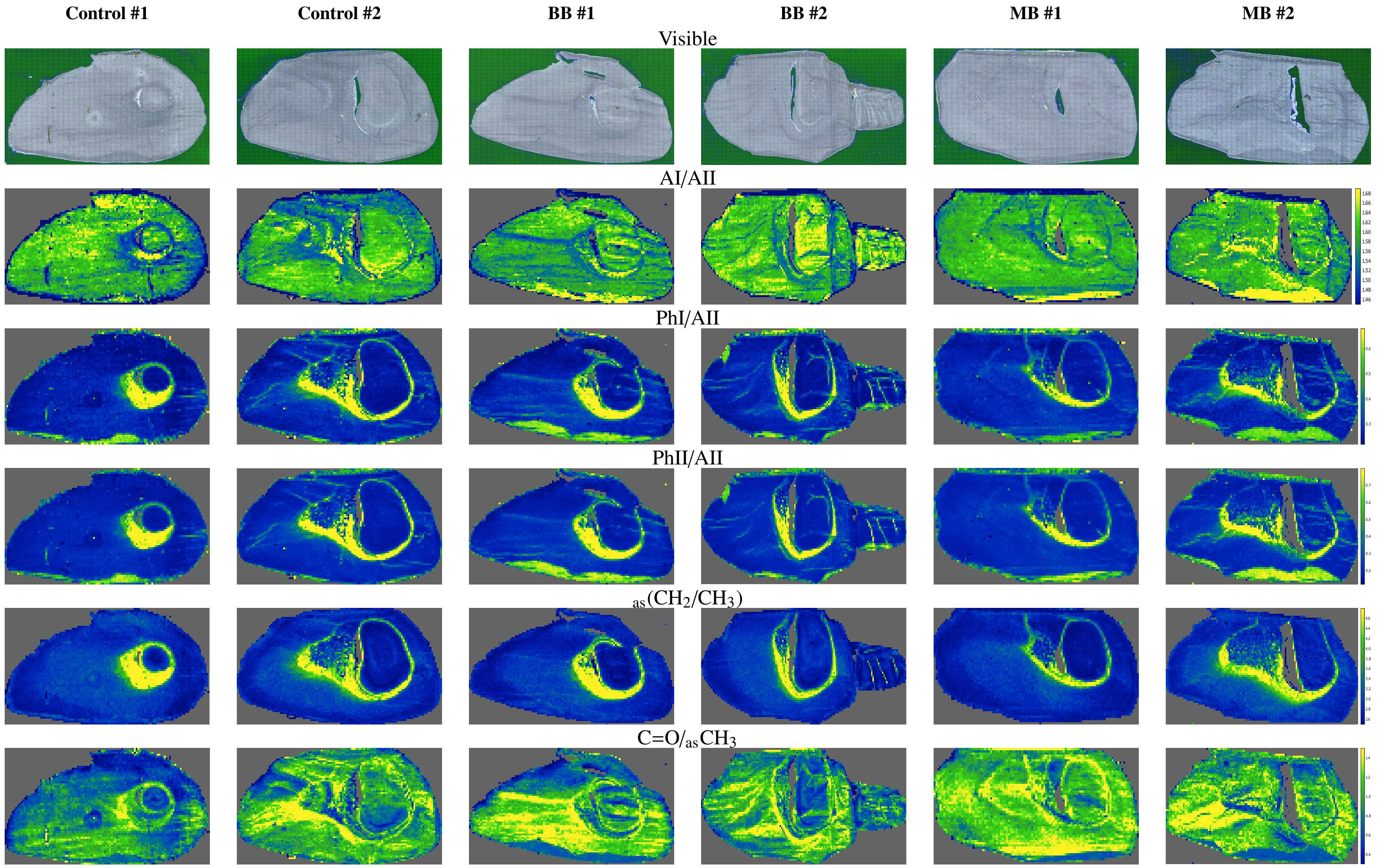

Figure S2. Optical (first row) and hyperspectral (rows 2–6) images of the healthy rat brain sections at 24 hours post-irradiations showing the distribution of the following spectral ratios (from top to bottom rows): AI/AII, PhI/AII, PhII/AII, $_{as}(CH_2/CH_3)$ and $C{=}O/_{as}CH_3$. Each column represents one animal and one irradiation configuration: control, broad beam (BB) and minibeams (MB). The mean dose for BB and pMBRT irradiations was 30 Gy. For pMBRT, the specific peak and valley doses were 59 ± 2 Gy and 14.5 ± 1.0 Gy. MBs were generated by means of a divergent collimator of 5 slits with a width of 400 µm, separated a c-t-c distance of 2.8 mm.

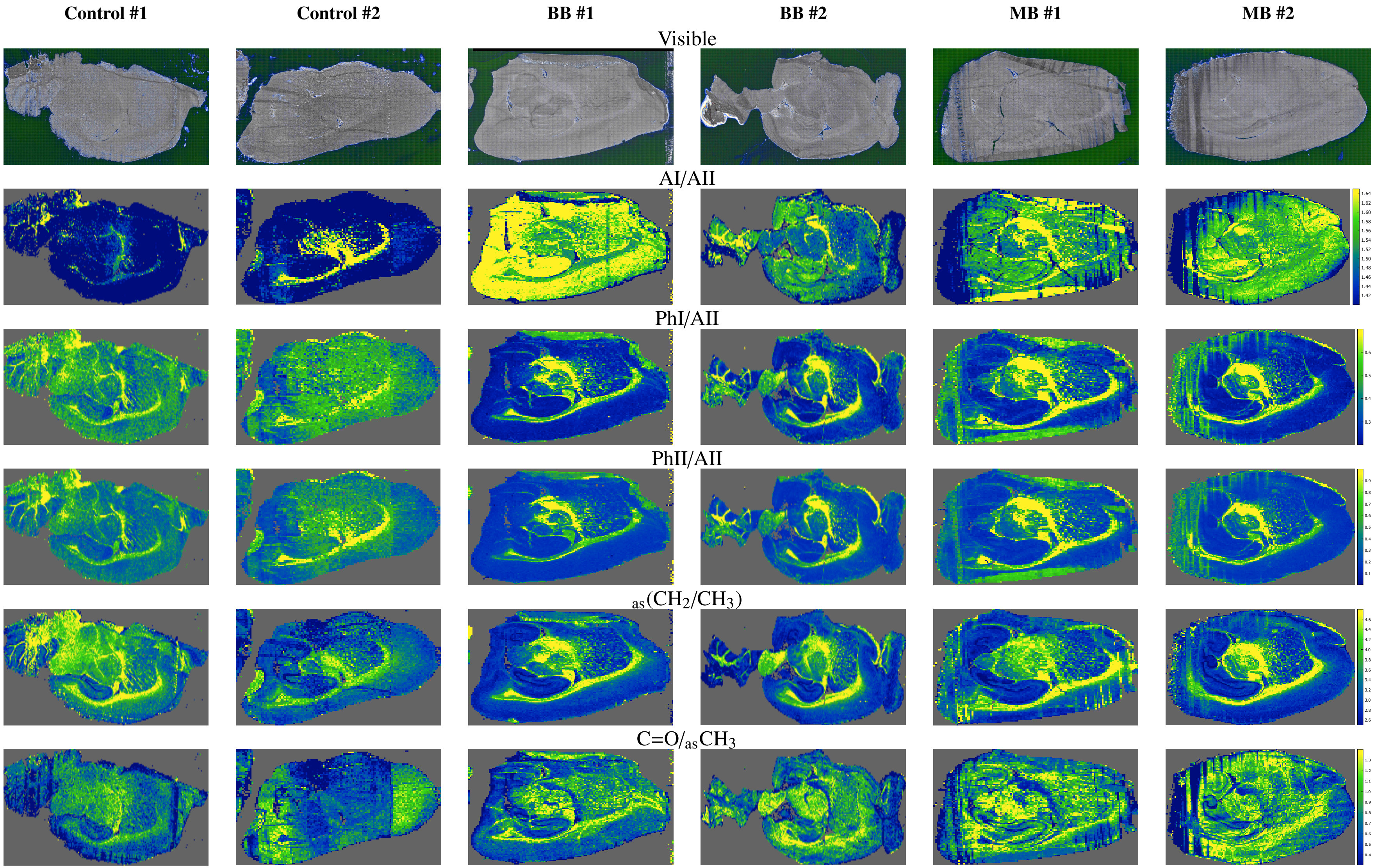

Figure S3. Optical (first row) and hyperspectral (rows 2–6) images of the healthy rat brain sections at 2 hours post-irradiations showing the distribution of the following spectral ratios (from top to bottom rows): AI/AII, PhI/AII, PhII/AII, $_{as}(CH_2/CH_3)$ and $C{=}O/_{as}CH_3$. Each column represents one animal and one irradiation configuration: control, broad beam (BB) and minibeams (MB). The mean dose for BB and pMBRT irradiations was 30 Gy. For pMBRT, the specific peak and valley doses were 59 ± 2 Gy and 14.5 ± 1.0 Gy. MBs were generated by means of a divergent collimator of 5 slits with a width of 400 µm, separated a c-t-c distance of 2.8 mm.

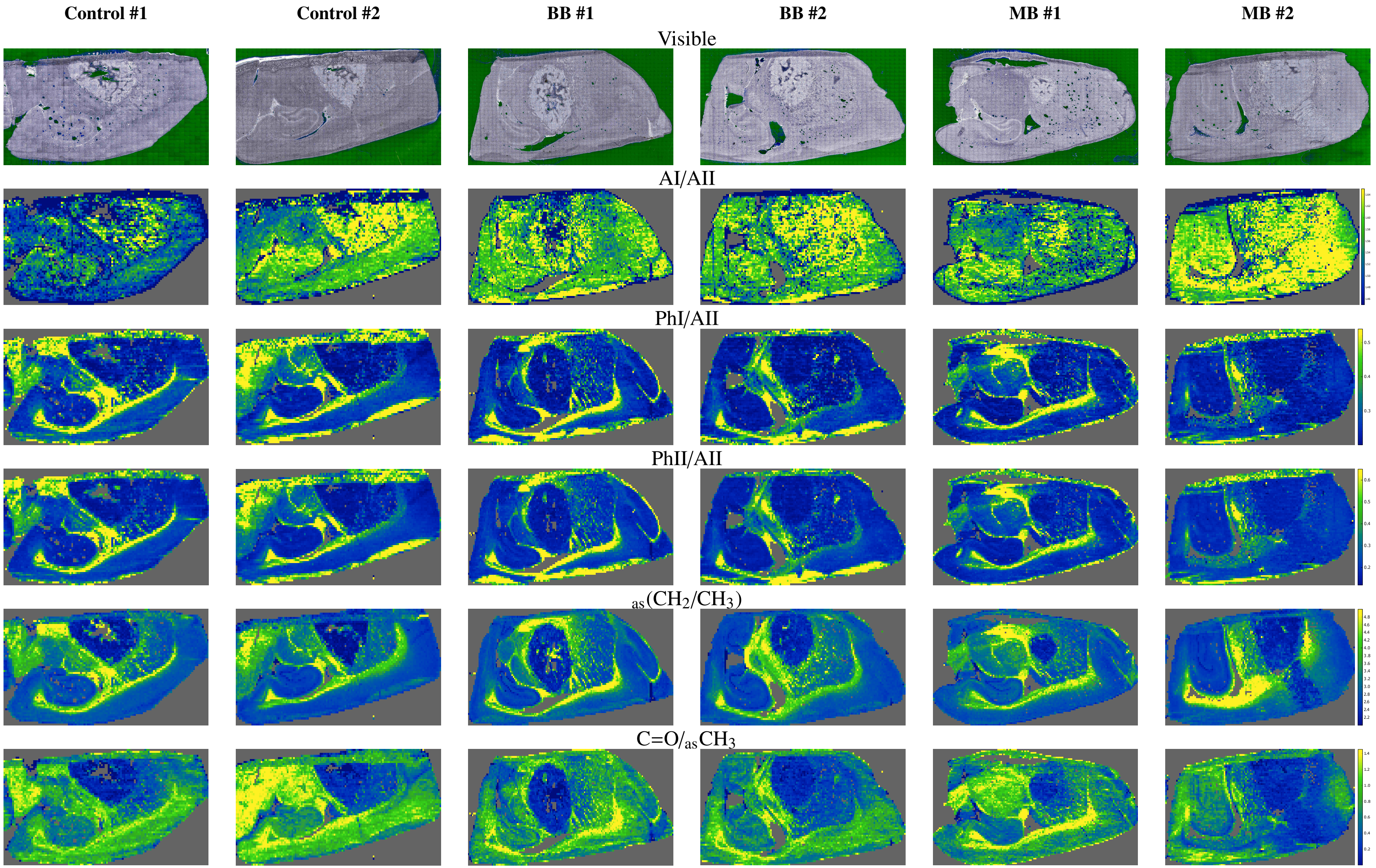

Figure S4. Optical (first row) and hyperspectral (rows 2–6) images of the tumour-bearing rat brain sections at 24 hours post-irradiations showing the distribution of the following spectral ratios (from top to bottom rows): AI/AII, PhI/AII, PhII/AII, $_{as}(CH_2/CH_3)$ and $C{=}O/_{as}CH_3$. Each column represents one animal and one irradiation configuration: control, broad beam (BB) and minibeams (MB). The mean dose for BB and pMBRT irradiations was 30 Gy. For pMBRT, the specific peak and valley doses were 59 ± 2 Gy and 14.5 ± 1.0 Gy. MBs were generated by means of a divergent collimator of 5 slits with a width of 400 µm, separated a c-t-c distance of 2.8 mm.
